# Dietary, Anthropometric, and Biochemical Determinants of Plasma High-Density Lipoprotein-Cholesterol in Free-Living Adults

**DOI:** 10.1155/2011/851750

**Published:** 2010-12-15

**Authors:** Erick Prado de Oliveira, Rodrigo Minoru Manda, Gabriel Augusto Torezan, José Eduardo Corrente, Roberto Carlos Burini

**Affiliations:** ^1^Department of Public Health, CeMENutri—Centre for Physical and Nutritional Metabolism, Sao Paulo State University (UNESP), District of Rubião Junior s/no, 18618-970 Botucatu, SP, Brazil; ^2^Department of Pathology, Sao Paulo State University (UNESP), District of Rubião Junior s/no, 18618-900 Botucatu, SP, Brazil; ^3^Department of Statistics, Sao Paulo State University (UNESP), District of Rubião Junior s/no, 18618-900 Botucatu, SP, Brazil

## Abstract

The level of high-density lipoprotein is thought to be critical in inhibiting lesion formation as well as reducing the lipid load of preexisting atherosclerotic lesions. With the aim of determining the main determinants of plasma HDL-cholesterol (HDL-c) in free-living adults, 997 individuals (52.3 ± 10 years, 67% females) were selected for a descriptive cross-sectional study. The used data corresponded to the baseline obtained from participants clinically selected for a lifestyle modification program. Covariables of clinical, anthropometry, food intake, aerobic fitness, and plasma biochemistry were analyzed against plasma HDL-c either as continuous or categorized variables. After adjustments for age, gender, and BMI the excess of abdominal fat along with high carbohydrate-energy intake and altered plasma triglycerides were the stronger predictors of reduced plasma HDL-c. In conclusion lifestyle interventions aiming to normalize abdominal fatness and plasma triglycerides are recommended to restore normal levels of HDL-c in these free-living adults.

## 1. Introduction

It is now established that oxidation of LDL constitutes a key event in inflammation and atherogenesis [1]. Mechanisms of LDL oxidation in vivo involve concerted modification through oxidation by oxidants produced by arterial wall cells, such as reactive nitrogen species, reactive chlorine species, hydroxyl radicals, and lipid soluble free radicals [2]. Such a spectrum of chemically diverse oxidants implies that any single low-molecular weight antioxidant such as vitamin E or C, even at physiologically relevant doses, may not provide complete oxidative protection of LDL *in vivo* [1, 2]. Atherogenic dyslipidemia is commonly characterized by elevated triglycerides, normal or slightly elevated LDL-c with a reduced HDL-c concentration [3]. Plasma high-density lipoprotein cholesterol (HDL-c) posses a spectrum of antiatherogenic actions, including potent antioxidant and anti-inflammatory activities [4]. The usual protective features of high HDL-c are reverse cholesterol transport, antioxidative, anti-inflammatory, antiapoptotic, antithrombotic, anti-infections, vasodilatation, and so forth [5].

Dietary and exercise modifications can lead to improvement of HDL-c concentrations, which may be associated with greater antioxidative role of HDL-c [6–9]. It has been demonstrated that HDL-c concentrations are positively associated with physical function in older adults [10].

In the ATTICA study, participants who where more physically active and consumed a diet closer to the Mediterranean type showed higher values of HDL-c. Furthermore women and those with lower BMI and younger age had higher HDL-c values [11].

The aim of this study was to determine the major determinants of plasma HDL-c in free-living adults clinically selected for a lifestyle modification program with physical exercises.

## 2. Methods

### 2.1. Individuals

A descriptive cross-sectional study was conducted in a subgroup of participants clinically screened for the lifestyle modification program “*Mexa-se Pró-Saúde* (Move for Health)”, from 2002 to 2009. This program is offered to patients with noncommunicable chronic diseases and consists of regular physical exercise and nutritional counseling. The Metabolism, Exercise and Nutrition Center (CeMENutri), conducts this program since 1992, in Botucatu. Botucatu is a city located in the center of Sao Paulo State, about 230 km west of the capital city and has a population of 121,274 in habitants [12]. 

The inclusion criteria for participants are individuals over the age of 35, of both genders, with at least one of the metabolic syndrome components and/or comorbities, and without metabolic or motor disabilities that would limit physical exercise. 

The 1129 individuals that attended the program during this period comprised 67% females and were 52.3 ± 10 years of age. All the subjects signed a free consent form, and the research project was approved by the Research Ethics Committee (document no. CEP 3271-2009) of the Botucatu School of Medicine (FMB), São Paulo State University (UNESP), Brazil.

**Figure 1:**
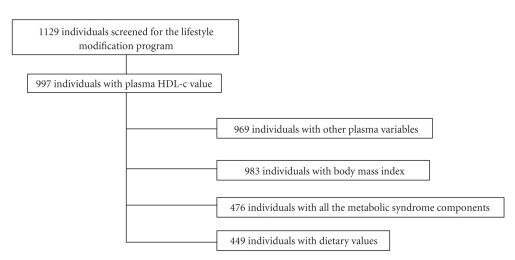
Flow-chart of study participation.

From those 1129 subjects, 997 had HDL-c data and were studied. Data on clinical, fitness, food consumption, body composition, and plasma biochemistry were available as indicated in the flow chart (Figure 1).

### 2.2. Dietary Intake

Usual dietary intake data was determined using a 24-hour recall. The diet was documented by trained personnel, and to obtain precise information, the subjects were asked how often they usually ate during the day, what variety of food was consumed, how the food was prepared, what the serving size was, and what the brand of the food/meal was. The diets were analyzed with the software NutWin (2002) version 1.5 [13], and the principal nutrients of interest were energy, protein, fat (saturated, mono- and polyunsaturated), cholesterol, carbohydrates, and dietary fiber. Mean individual nutrient intakes per day were computed using the NutWin database and Brazilian food tables [14–16]. The Healthy Eating Index (HEI) modified for the Brazilian population was used to assess the quality of the participant's diet [17]. The original HEI was developed based on a 10-component system of five food groups with a total possible index score of 100. This method was adapted for the Brazilian population based on the Brazilian food guide that has eight food groups and 12 components to measure the variety of food intake. Each of the 12 components has a score ranging from 0 to 10, so the total possible index score is 120.

### 2.3. Anthropometry

Body weight was measured by a platform-type anthropometric scale (Filizola) with a maximum capacity of 150 kg and an accuracy of 0.1 kg. Height was determined by a portable Seca stadiometer with an accuracy of 0.1 cm [18]. Using body weight and height measurements, BMI (weight/height (m^2^) was calculated.

Waist circumference (WC) was measured at the point midway between the last rib and the iliac crest. A steel Sanny anthropometric tape measure (without a lock) was used for all measurements. 

A bioelectrical impedance device (Biodynamics, model 450, USA) was used to determine body fat percentage (%BF) [19] and body-muscle mass whose data were used for the muscle-mass index (MMI) calculation [20].

### 2.4. Biochemical Analyses

Blood samples were collected by vacuum venous puncture, after a 10- to 12-hour fasting period. The individuals were previously advised to not perform vigorous physical exercises 24-hours and/or consume alcohol 72-hours prior to blood collection. Laboratory analysis was performed within 4 hours after blood collection using the dry-chemistry method (Vitros system, Johnson & Johnson), plasma ultrasensititivity C-reactive protein (CRP) was measured by immulite kit (DPC) Medlab-diagnostic Products Corporation, Los Angeles, CA.

### 2.5. Cardiorespiratory Index (VO_2max_)

The VO_2max_ was indirectly measured through the time spent in electrical treadmill [21] during the Balke protocol [22].

### 2.6. Operational Definition of Variables

Overweight was classified as BMI ≥25 kg/m^2^ [23], altered WC was considered when above 102 cm (40.16 inches) for men and above 88 cm (34.65 inches) for women [24]. Sarcopenia was defined by muscle mass index (MMI) lower than 10.75 kg/m^2^ for men and below 6.75 kg/m^2^ for women [25]. Higher body fat was defined by body fatness higher than 25% for men and higher than 35% for women [26]. Hypertriglyceridemia was defined by plasma concentrations ≥150 mg/dL [27], lower HDL-c as <40 mg/dL for men and <50 mg/dL for women [27] and hypercholesterolemia was >200 mg/dL [28]. Higher plasma glucose was defined by ≥100 mg/dL, higher CRP by ≥0.3 mg/dL, and higher uric acid when in the 4th quartile.

### 2.7. Statistical Analysis

Statistical analyses were conducted with SAS software for windows (SAS version 9.1.3., SAS Institute, Inc., Cary, NC). Descriptive statistics were performed for the study and continuous variables are presented as means ± standard deviation (SD). Continuous variables were compared by the Wilcoxon test. Pearson correlation was applied to observe the correlationship between HDL-c and body composition, dietary consumption, and biochemistry analysis.

Regression models for data with negative binomial distribution were fitted for food intake characterization. In order to determine the probability of HDL-c alteration by food intakes, anthropometry, and biochemical analysis, *P* < .05 was adopted as a significant value.

## 3. Results

Reduced plasma HDL-c levels were found in individuals with higher BMI, WC, and body fat, higher plasma concentrations of uric acid, triglycerides, and CRP along with lower values of muscle-mass index, VO_2max_ and plasma albumin (Table 1).

**Figure 2:**
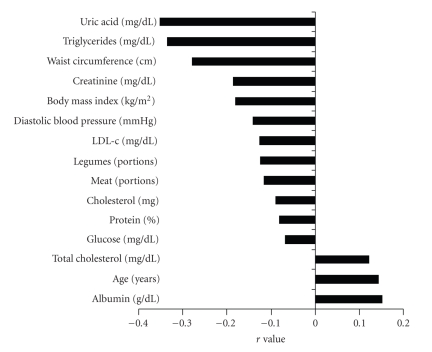
Statistical significant correlationships (*P* < .05) found for plasma HDL-c concentrations.

Plasma HDL values correlated positively (*P* < .05) with age, total cholesterol, and albumin. They were negatively correlated (*P* < .05) with BMI, WC, ingested (%) protein, cholesterol (mg/day), serving of legumes and meats, plasma uric acid, LDL-c, glucose, TG, total protein, and DBP (Figure 2). There was no significant correlationship between plasma HDL-c and the variables: body fat (%), MMI (kg/m^2^), the energy contribution of ingested CHO, total and saturated, mono- or polyunsaturated fat, ingested fiber, and servings of grains, fruits, dairy, sugar, and oils, as well as dietary variety and HEI. The correlationship was not significant also for values of plasma, CRP, and SBP.

Table 2 contains the odds ratio of reduced HDL-c in function of abnormal anthropometry.  Table 3 contains the odds ratio for changed plasma variables and Table 4 for the altered intake of dietary components. After adjustments for sex, age, and BMI the waist circumference, along with high CHO-energy intake and elevated plasma levels of triglycerides were the stronger determinants of reduced plasma HDL-c, whereas higher plasma cholesterol presented a protective effect. By using the other four components of metabolic syndrome (blood hypertension, waist circumference and plasma triglycerides and glucose) for adjustments the resulted odds ratio presented plasma triglycerides and waist circumference as risk factor and plasma cholesterol as a protective factor against low HDL-c plasma.

## 4. Discussion

From the dietary intake only meat, cholesterol, and legumes correlated (negatively) significantly with plasma HDL-c concentrations. Differently of these data others had revealed that dietary cholesterol increases the HDL-c level in women [11]. Moreover they described that polyunsaturated fat and the polyunsaturated/saturated lipid ratio decrease the HDL-c level. Presently, we found that HDL-c was decreased by intaking higher serving of meat, and also by the higher protein-energy contribution. However, after the adjustments for gender, age, BMI, and total energy intake (TEI) the higher CHO intake was the only dietary risk factor found for abnormal HDL-c. 

Many recommendations have been provided to the public to reduce dietary fat, with some claims that health benefit will be achieved [29]. However in human studies when total fat is replaced by carbohydrate intake, the result is a decreased plasma concentration of fasting HDL-c and elevated triglycerides, which may counteract the benefit of lowering LDL-c. Therefore increasing carbohydrate intake may adversely affect lipoprotein concentrations as seen in the present work. Park et al. also found in Korean women that higher carbohydrate intakes were significantly associated with low HDL-c levels [30]. Additionally, significantly lower HDL-c were associated with sugar consumed by high BMI African American children [31].

In 2006, the American Heart Association revised their diet and lifestyle recommendations adding a recommendation to minimize intakes of beverages and foods with added sugars [32].

Among the other lifestyle contributions to HDL-c levels [11] no correlationships were found for VO_2max _ and smoking (plasma SCN levels), the alcohol consumption was not assessed. 

In a more comprehensive study (ATTICA study) Chrysohoou et al. [11] found that participants who were more physically active and consumed a diet closer to the Mediterranean type showed higher values of HDL-c. In the present data neither diet quality (HEI)n or VO_2max _ proved that the studied sample was either physically active or on Mediterranean-style diet.

As already referred by others [11] subjects with lower BMI had higher HDL-c levels. In the present data not only BMI but all other markers of hyperadiposity were predictors of reduced HDL-c levels. From those markers only WC remained as independent risk factors for lower HDL-c.

Changed plasma values of uric acid, triglycerides, and CRP were significant risk factors for altered HDL-c even after gender and age adjustments. However, only high plasma uric acid and triglycerides persisted after the BMI adjustment. Furthermore only triglycerides represented an independent plasma risk factor for altered HDL-c after adjustments including the remaining components of metabolic syndrome.

Thus, the found independent risk factors for reduced HDL-c were higher dietary energy contribution of CHO, higher WC, and higher plasma triglycerides.

In the present work higher CRP functioned as a risk factor for lower HDL-c until the adjustment for BMI. Obesity is known as an inflammatory disease [33] which might decrease HDL-c concentrations [34]. 

The present data shows altered uric acid as a predictor of reduced HDL-c. Higher uric acid and low-grade inflammation are associated with impaired lipoprotein metabolism even before clinically visible symptoms of atherosclerosis are apparent [3].

Uric acid may function as a potent antioxidant by scavenging free radicals and also by stabilizing ascorbate in biological fluids [35]. Therefore elevated uric acid could be considered to be a compensatory mechanism that counteracts oxidative stress related to the preponderance of impaired HDL particles [3].

The main limitation of our study resides in its cross-sectional design. Given the study design, we could not investigate any cause-effect mechanism for the studied relationships but only generate hypothesis. Secondly, the study sample size was relatively small which could limit the generalization of our results. Finally, the fact that the subjects volunteered for the lifestyle changing program could have introduced a selection bias. Also we cannot completely rule out possible inaccuracy in one-day 24 hour-food intake recall.

## 5. Conclusions

This study highlights the inverse relationship between HDL-c levels and higher intake of carbohydrates, along with excess of abdominal adiposity and higher plasma levels of triglycerides. These associations may confer a further prescription of lifestyle changing program for improving dietary intake, physical fitness, and body fatness.

##  Conflict of Interest 

The authors declare that they have no conflict of interest.

## Figures and Tables

**Table 1:** Demographic, anthropometric, clinical, and laboratory characterization of individuals with normal and abnormal high density lipoprotein cholesterol.

	*n*	Normal HDL	*n*	Abnormal HDL	*P*
Age (years)	617	53.5 ± 9.8	366	52.5 ± 10.3	.09
Body Mass Index (kg/m^2^)	617	28.7 ± 5.3	366	30.0 ± 5.1	<.0001
Waist Circumference (cm)	613	93.9 ± 13.8	366	97.5 ± 12.8	<.0001
Body Fat (%)	472	31.4 ± 8.0	303	33.3 ± 8.9	.005
Muscle Mass Index (kg/m^2^)	380	10.9 ± 5.1	233	9.55 ± 4.5	.0004
Total Energy Intake (kcal)	615	1627 ± 604	371	1562± 614	.08
Carbohydrate (%)	615	51.2 ± 10.2	371	51.5 ± 10.1	.25
Protein (%)	615	18.5 ± 5.9	371	18.8 ± 5.9	.59
Fat (%)	615	30.3 ± 8.8	371	29.6 ± 8.6	.14
Saturated Fat (%)	283	8.7 ± 4.1	253	8.9 ± 3.9	.52
Monounsaturated Fat (%)	283	9.2 ± 3.6	253	9.1 ± 3.7	.63
Polyunsaturated Fat (%)	283	6.8 ± 3.2	253	6.5 ± 3.3	.20
Cholesterol (mg/day)	553	191 ± 142	329	193± 134	.56
Fiber (servings)	614	13.7 ± 7.3	369	13.5 ± 7.8	.30
Cereal (servings)	235	3.6 ± 2.0	221	3.5 ± 1.7	.58
Fruits (servings)	235	2.5 ± 2.8	221	2.2 ± 2.6	.12
Vegetables (servings)	235	2.6 ± 3.4	221	2.4 ± 2.5	.45
Legumes (servings)	235	1.1 ± 1.5	221	1.1 ± 1.5	.27
Dairy (servings)	235	1.4 ± 1.3	221	1.3 ± 1.2	.64
Meat (servings)	235	2.0 ± 1.7	221	1.9 ± 1.5	.83
Sugar (servings)	235	1.6 ± 1.9	221	1.5 ± 1.8	.63
Oil (servings)	235	2.5 ± 2.1	221	2.7 ± 7.9	.19
Variety (servings)	235	13.1 ± 4.2	221	12.6 ± 4.2	.22
Health Eat Index	235	80.3 ± 15.8	221	79.1 ± 14.8	.37
Plasma urea (mg/dL)	609	32.4 ± 10.4	365	32.6 ± 12.5	.74
Plasma creatinine (mg/dL)	612	0.94 ± 0.25	366	0.96 ± 0.21	.11
Plasma uric Acid (mg/dL)	609	4.53 ± 1.61	360	5.11 ± 1.48	<.0001
Plasma LDL-c (mg/dL)	580	127 ± 35.4	333	132± 37.0	.09
Plasma glucose (mg/dL)	612	101 ± 29.9	365	103± 35.1	.15
Plasma total Cholesterol (mg/dL)	614	216 ± 41.2	366	207± 39.9	.03
Plasma triglycerides (mg/dL)	584	142 ± 79.4	324	197± 140.61	<.0001
Plasma total Protein (g/dL)	548	7.3 ± 0.58	289	7.4 ± 0.66	.002
Plasma albumin (g/dL)	572	4.5 ± 0.5	313	4.3 ± 0.5	<.0001
Plasma c-Reactive Protein (mg/dL)	151	0.36 ± 0.46	105	0.58 ± 0.75	.001
VO_2max _	69	37.3 ± 9.8	45	33.3 ± 10.0	.03
Systolic blood pressure (mmHg)	342	128 ± 18.3	142	129± 15.4	.18
Diastolic blood pressure (mmHg)	342	81.1 ± 11.5	142	82.7 ± 10.4	.13

**Table 2:** Logistic regression analysis for the association of reduced plasma HDL-c concentration with the anthropometric variables.

	Model 1	Model 2	Model 3
Waist Circumference (abnormal versus normal)	1.697 (1.305–2.207)	1.712 (1.308–2.242)	1.40 (1.02–1.91)
Body Mass Index (abnormal versus normal)	1.901 (1.347–2.680)	1.941 (1.367–2.754)	—
% Body Fat (abnormal versus normal)	1.398 (1.044–1.872)	1.436 (1.068–1.930)	1.24 (0.90–1.71)
Muscle Mass Index (abnormal versus normal)	1.113 (0.788–1.572)	1.190 (0.836–1.692)	1.42 (0.90–2.07)

Model 1 = crude

Model 2 = adjusted for sex and age

Model 3 = adjusted for model 2 + Body Mass Index.

**Table 3:** Logistic regression analysis for the association of reduced plasma HDL-c concentration with the other blood markers.

	Model 1	Model 2	Model 3	Model 4
Urea (abnormal versus normal)	1.097 (0.790–1.522)	1.152 (0.821–1.615)	1.166 (0.829–1.642)	0.940 (0.510–1.736)
Creatinine (abnormal versus normal)	1.692 (0.726–3.952)	1.821 (0.776–4.273)	1.736 (0.735–4.098)	1.057 (0.238–4.672)
Uric Acid (abnormal versus normal)	1.879 (1.302–2.717)	1.945 (1.335–2.832)	1.715 (1.164–2.525)	2.036 (0.995–4.166)
Glucose (abnormal versus normal)	0.933 (0.710–1.228)	0.968 (0.730–1.282)	0.879 (0.660–1.173)	0.638 (0.401–1.017)
Triglycerides (abnormal versus normal)	2.645 (1.996–3.496)	3.030 (2.257–4.081)	2.801 (2.070–3.787)	2.816 (1.776–4.464)
Albumin (abnormal versus normal)	0.729 (0.140–3.787)	0.689 (0.131–3.597)	0.710 (0.135–3.717)	—
C-Reactive Protein (abnormal versus normal)	1.769 (1.070–2.932)	1.706 (1.018–2.857)	1.367 (0.794–2.358)	1.689 (0.747–3.816)
Total Cholesterol (abnormal versus normal)	0.643 (0.492–0.840)	0.664 (0.506–0.872)	0.656 (0.498–0.864)	0.453 (0.278–0.736)
LDL-c (abnormal versus normal)	1.035 (0.790–1.355)	1.094 (0.832–1.438)	1.091 (0.828–1.436)	1.109 (0.708–1.739)

Model 1 = crude

Model 2 = adjusted for sex and age

Model 3 = adjusted for model 2 and Body Mass Index

Model 4 = adjusted for model 3 and systolic blood pressure, diastolic blood pressure, waist circumference, triglycerides, glucose.

**Table 4:** Logistic regression analysis for the association of reduced plasma HDL-c concentration with the dietary intake parameters.

	Model 1	Model 2
% CHO (≥50% VERSUS <50%)	1.258 (0.958–1.636)	1.373 (1.046 – 1.803)
% Protein (≤15% VERSUS >15%)	0.916 (0.689–1.218)	0.982 (0.731–1.318)
% Fat (<35% VERSUS ≥35%)	1.188 (0.883–1.599)	1.238 (0.907–1.690)
% Saturated fat (≤10 VERSUS >10%)	1.045 (0.731–1.493)	1.082 (0.747–1.568)
% Monounsaturated fat (≤20% VERSUS >20%)	1.799 (0.327–9.906)	1.876 (0.311–10.642)
% Polyunsaturated fat (≤10 VERSUS >10%)	1.229 (0.740–2.040)	1.183 (0.705–1.984)
Cholesterol (<200 mg VERSUS >200 mg)	1.005 (0.683–1.478)	1.012 (0.666–1.536)
Cereals (≥5 VERSUS <5 servings)	0.861 (0.540–1.374)	1.129 (0.665–1.916)
Fruits (≥3 VERSUS <3 servings)	0.845 (0.574–1.246)	0.903 (0.604–1.351)
Vegetables (≥4 VERSUS <4 servings)	1.041 (0.665–1.620)	1.033 (0.643–1.653)
Legumes (≤2 VERSUS >2 servings)	1.123 (0.777–1.621)	1.223 (0.823–1.816)
Dairy (≥3 VERSUS <3 servings)	0.911 (0.529–1.569)	1.100 (0.563–1.812)
Meat (≤2 VERSUS >2 servings)	0.988 (0.678–1.470)	0.905 (0.583–1.400)
Oil (≤2 VERSUS >2 servings)	0.728 (0.499–1.061)	0.797 (0.521–1.220)
Variety (≥8 VERSUS <8)	0.697 (0.288–1.688)	0.803 (0.326–1.976)
Fiber (≥20 g VERSUS <20 g)	0.988 (0.708–1.406)	1.168 (0.810–1.682)
Health Eat Index (>100 VERSUS ≤100 points)	0.768 (0.399–1.480)	0.664 (0.330–1.337)

Model 1 = crude

Model 2 = adjust for sex, age, total energy intake and body mass index.
